# A Putative Gene Cluster from a *Lyngbya wollei* Bloom that Encodes Paralytic Shellfish Toxin Biosynthesis

**DOI:** 10.1371/journal.pone.0014657

**Published:** 2011-02-10

**Authors:** Troco K. Mihali, Wayne W. Carmichael, Brett A. Neilan

**Affiliations:** 1 School of Biotechnology and Biomolecular Sciences, The University of New South Wales, Sydney, Australia; 2 Department of Biological Sciences, Wright State University, Dayton, Ohio, United States of America; 3 Australian Centre for Astrobiology, The University of New South Wales, Sydney, Australia; Griffith University, Australia

## Abstract

Saxitoxin and its analogs cause the paralytic shellfish-poisoning syndrome, adversely affecting human health and coastal shellfish industries worldwide. Here we report the isolation, sequencing, annotation, and predicted pathway of the saxitoxin biosynthetic gene cluster in the cyanobacterium *Lyngbya wollei*. The gene cluster spans 36 kb and encodes enzymes for the biosynthesis and export of the toxins. The *Lyngbya wollei* saxitoxin gene cluster differs from previously identified saxitoxin clusters as it contains genes that are unique to this cluster, whereby the carbamoyltransferase is truncated and replaced by an acyltransferase, explaining the unique toxin profile presented by *Lyngbya wollei*. These findings will enable the creation of toxin probes, for water monitoring purposes, as well as proof-of-concept for the combinatorial biosynthesis of these natural occurring alkaloids for the production of novel, biologically active compounds.

## Introduction

Saxitoxin and its derivatives, collectively termed paralytic shellfish toxins (PST), are a group of low molecular weight, highly potent neurotoxic alkaloids which inhibit nerve conduction and muscle contraction by selectively binding and blocking of sodium channels. PSTs are the causative agents of the syndrome termed paralytic shellfish poisoning (PSP) [1], [2], [3]. Saxitoxin (STX), a tricyclic perhydropurine alkaloid, is considered the parent compound of the PSTs and may be substituted at five positions, leading to more than 30 naturally occurring analogs [4], [5], [6], [7], [8], [9]. PSP is a life-threatening affliction with a worldwide distribution, mainly caused by the consumption of shellfish contaminated by PSTs. PSP outbreaks have prompted serious public health concerns as well as significant economic losses due to the closure of fisheries, effects on tourism and costly toxin monitoring programs [10]. The human vectors of PSP are not the primary producers of PSTs, but have been shown to bio-accumulate them via filter feeding on toxic marine dinoflagellates mainly belonging to the genera *Alexandrium, Gymnodinium* and *Pyrodinium* [11], [12], [13]. Interestingly, PST biosynthesis has also been identified in several genera of freshwater cyanobacteria, namely *Anabaena*, *Cylindrospermopsis*, *Aphanizomenon, Planktothrix*, *Raphidiopsis brookii* and *Lyngbya wollei* [14], [15], [16], [17], [18], [19]. Furthermore, early investigations into PST biosynthesis mechanisms in cyanobacteria and dinoflagellates have indicated that they are synthesised via the same biosynthetic route [20]. Cyanobacteria present a formidable challenge for water management industries, due to toxic and non-toxic strains often being closely related and frequently indistinguishable using traditional taxonomic approaches. The combination of unpredictable formation of dense toxic blooms in water reservoirs and inability to identify toxic strains requires the use of time and resource consuming analytical methods such as HPLC and LC-MS to identify toxins in the water column. Genetic probes specific to toxin biosynthesis genes can afford an early warning system to help manage and control these toxic cyanobacterial blooms, also referred to as Harmful Algal Blooms (HABs).

We have recently identified and characterized putative PST biosynthesis gene clusters in the cyanobacteria *Cylindrospermopsis raciborskii* T3, *Anabaena circinalis* AWQC131C and *Aphanizomenon* sp. NH-5 [21], [22]. We have been able to show that the gene clusters are similar in gene content, though several putative tailoring enzymes are variable, leading to indirect evidence of the role of the tailoring gene *sxtX*. The *Anabaena* and *Aphanizomenon* PST gene clusters are apparently more similar in gene content and cluster organization to each other than to the initially characterized PST gene cluster in *C. raciborskii*, indicating the gene cluster is most probably of ancient origin, and shares a common descent among these closely related species, while the sporadic distribution of toxicity in the genera is explained through the repeated losses of the biosynthetic cluster. Following the lines of that work, we set out to characterize and identify the PST gene cluster in *L. wollei,* which has been shown to produce novel PST analogs, while lacking more commonly found carbamoylated PST derivatives. In addition, the production of various PST congeners appears to be dependent on the producing species and its geographical origin [7], [9], [23], [24]. This variability in toxin production has a presumable origin in the gene content of the PST gene cluster, its elucidation would thereby provide further insight into the PST biosynthetic machinery and the evolution of this intriguing gene cluster.

**Figure 1:**
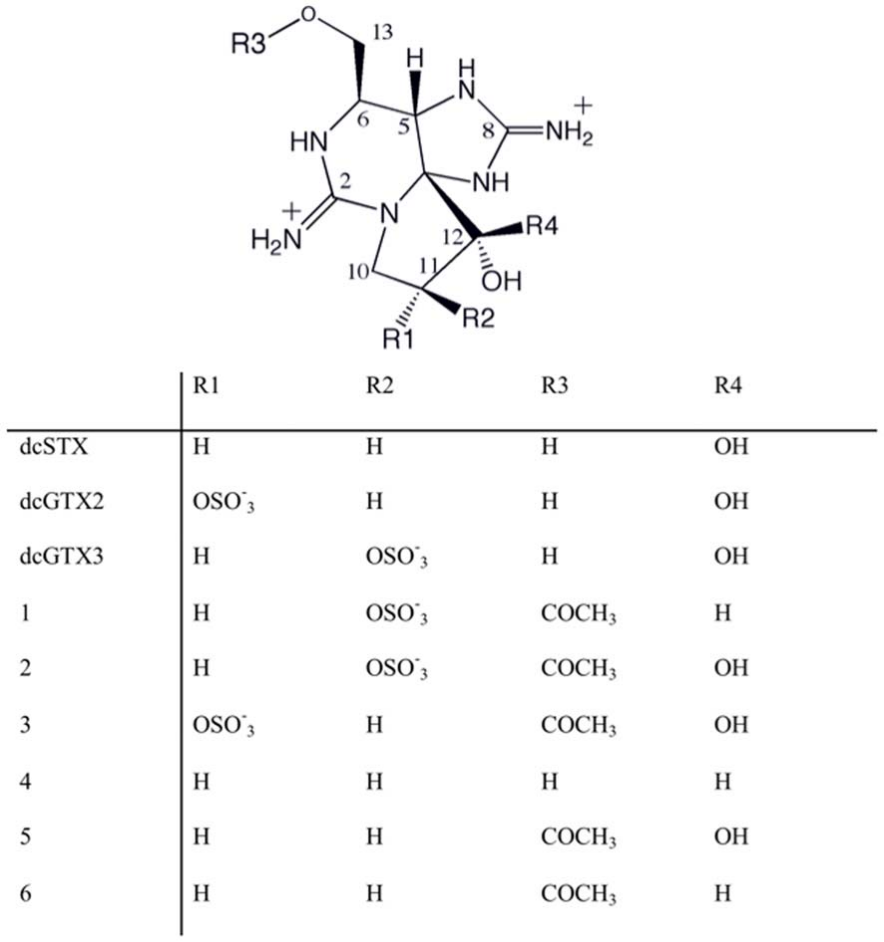
Structure of the saxitoxin analogs identified in *Lyngbya wollei* (adapted from [7]).

*Lyngbya wollei* a cyanobacterium belonging to the cyanobacterial order *Oscillatoriales,* has been reported from south-eastern United States lakes and water reservoirs for the past 100 years and has become increasingly common in the last three decades [25]. *L. wollei* has been shown to produce various PSTs, including decarbamoyl saxitoxins (dcSTX) and decarbamoyl gonyautoxins (dcGTX2, dcGTX3), as well as six novel analogs denoted **1-6** (Figure 1), which are characterized by the presence of acetate at C-13 and a carbinol at C-12 [7]. The toxin profile of *L. wollei* does not resemble the profile of any other characterized PST-producing cyanobacteria, such as *Anabaena circinalis, Aphanizomenon sp.* and *Cylindrospermopsin raciborskii*. The major PSTs found in *A. circinalis* are STX, the GTXs, and C1 and C2 [15], [26], while the major PST toxins found in *Aph. flos-aquae* (*Aph.* sp. NH-5) are neoSTX and STX [14].

Here we report the identification and sequencing of a homologous PST gene cluster in *L. wollei*. We present the bioinformatically inferred functions for most of the open reading frames (ORFs) in this gene cluster. The identification of saxitoxin biosynthesis gene sequences in various producer organisms enables the creation of probes for the monitoring of toxic blooms.

## Results and Discussion

### Identification of the Lyngbya PST gene cluster

**Figure 2:**
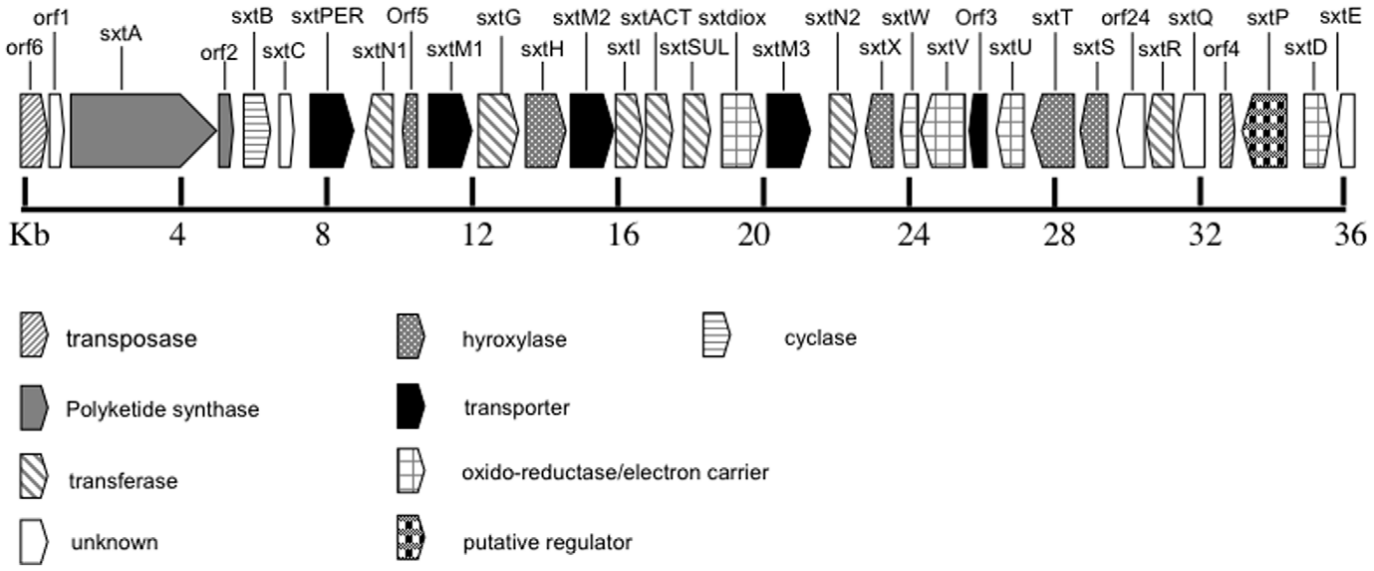
Organization of the PST biosynthesis cluster in *Lyngbya wollei*, scale indicates gene size in base pairs, direction of arrowed boxes indicates direction of transcription.

Using the degenerate PCR described, a single amplicon of 400 bp was amplified, cloned and sequenced from *L. wollei* genomic DNA. These gene fragments represented good candidates for saxitoxin biosynthesis genes due to their homology to the saxitoxin genes (*sxtT* and *sxtH*) previously identified in *C. raciborskii* T3 [21]. Numerous rounds of gene-walking outwards from these known regions revealed a SXT gene cluster in *L. wollei* spanning approximately 36 kb (Figure 2) and encoding thirty-one genes predicted to be involved in the biosynthesis and export of the these neurotoxins (Table 1). Homology analysis of the regions flanking the PST biosynthetic cluster confirmed that the sequences obtained are most related to *Lyngbya* species.

**Table 1:** The saxitoxin biosynthesis genes in *Lyngbya wollei* and their homology-based functions identified using BlastX (percentage identity is reported).

Gene	Size (bp)	Closest BLAST match (%)		Putative Function
Orf6	1009	ABZ02176.1 *Planktothrix agardhii* NIVA-CYA 126/8	66	Transposase/frame shift/inactive
Orf 1	240	No significant similarity	---	Unknown
sxtA	3732	ACG63801.1 SxtA *Aphanizomenon* sp. NH-5	90	Loading of ACP, Methylation, ACP, Claisen condensation
Orf 2	112	ABX60161.1 CyrB (AoaB) *C. raciborskii* AWT205 *	61	Truncated/inactive
sxtB	969	ACG63800.1 SxtB *Aphanizomenon* sp. NH-5	91	Cyclization
sxtC	285	ABI75092.1 SxtC *Cylindrospermopsis raciborskii* T3	78	Unknown
sxtPER	1218	ABI75130.1 SxtPER *Anabaena circinalis* AWQC131C	89	Export of PST's
sxtN1	837	ABI75104.1 SxtN *Cylindrospermopsis raciborskii* T3	93	Sulfotransfer
Orf 5	75	ACG63814.1 SxtH *Aphanizomenon* sp. NH-5	92	Truncated/inactive
sxtM 1	1440	ACG63815.1 SxtM *Aphanizomenon* sp. NH-5	81	Export of PSTs
sxtG	1134	ABI75136.1 SxtG *Anabaena circinalis* AWQC131C	94	Amidinotransfer
sxtH	1029	ABI75098.1 SxtH *Cylindrospermopsis raciborskii* T3	81	C-12 hydroxylation
sxtM 2	1458	ACG63815.1 SxtM *Aphanizomenon* sp. NH-5	80	Export of PSTs
sxtI	1071	ACC69003.1 SxtI *Cylindrospermopsis raciborskii* T3	80	Truncated/inactive
sxtACT	1197	ZP_05376006.1 *H. denitrificans* ATCC51888	33	C13 acylation
sxtSUL	909	CAJ70870.1 Candidatus *Kuenenia stuttgartiensis*	35	Sulfotransfer
sxtdiox	1005	ACG63810.1 SxtT *Aphanizomenon* sp. NH-5	83	C-12 reduction
sxtM 3	1512	ACG58379.1 SxtM *Anabaena circinalis* AWQC131C	88	Export of PSTs
sxtN 2	837	ABI75104.1 SxtN *Cylindrospermopsis raciborskii* T3	94	Sulfotransfer/inactive
sxtX	774	ACF94656.1 SxtX *Cylindrospermopsis raciborskii* T3	98	N-1 hydroxylation
sxtW	330	ABI75106.1 SxtW *Cylindrospermopsis raciborskii* T3	100	Electron carrier
sxtV	1680	ABI75107.1 SxtV *Cylindrospermopsis raciborskii* T3	95	Dioxygenase reductase
Orf 3	358	ABI75130.1 SxtPER *Anabaena circinalis* AWQC131C	83	Truncated/inactive
sxtU	750	ABI75108.1 SxtU *Cylindrospermopsis raciborskii* T3	92	Reduction of C-1
sxtT	1005	ACG63810.1 SxtT *Aphanizomenon* sp. NH-5	90	C-12 hydroxylation
sxtS	801	ABI75110.1 SxtS Cylindrospermopsis raciborskii T3	89	Ring formation
Orf 24	747	ABI75131.1 Orf24 *Anabaena circinalis* AWQC131C	82	Unknown
sxtR	777	ABI75112.1 SxtR *Cylindrospermopsis raciborskii* T3	94	Unknown
stxQ	777	ACG63806.1 SxtQ *Aphanizomenon* sp. NH-5	93	Unknown
Orf 4	279	ACC85294.1 transposase *Nostoc punctiforme* PCC73102	67	Transposition/inactive
sxtP	1482	ABI75126.1 SxtP *Anabaena circinalis* AWQC131C	86	Regulator/pilli formation
sxtD	759	ABI75125.1 SxtD *Anabaena circinalis* AWQC131C	85	Desaturation
sxtE	363	ABI75124.1 SxtE *Anabaena circinalis* AWQC131C	90	Unknown

*Indicates a PsiBLAST search was used.

### Characterization of the PST gene cluster

*L. wollei* possesses a toxin profile that is markedly different to that of other known PST producers. The production of these novel saxitoxin analogs, that are either decarbamoylated, acetylated, or hydroxylated (Figure 1) is hypothesized to be the result of the comparative differences between the genetic background of *L. wollei* and other cyanobacterial producers of PSTs. The putative saxitoxin gene cluster in *L. wollei* is highly similar in gene content, however, it includes additional enzymes not present, in the so far characterized PST biosynthetic clusters [19], [21], [22]. Specifically, an extra dioxygenase (*sxtdiox*, present in *R. brookii*), two additional sulfotransferases (*sxtN2* and *sxtSUL* (present in *R. brookii*), two additional exporters (*sxtM1* and *sxtM2*), and a novel acyl transferase (*sxtACT*) define the *L. wollei* pathway. Furthermore, the *L. wollei* PST gene cluster encodes a truncated carbamoyltransferase (*sxtI*) and does not contain the gene *sxtL,* coding for a GDSL-lipase-like enzyme identified in the other recently characterized saxitoxin gene clusters [19], [21], [22]).

Bioinformatic analysis of the *L. wollei* saxitoxin gene cluster further revealed a putative recombination event, which presumably results in the synthesis of the novel C-13 acetate saxitoxin analogs, and accounts for the lack of any analogs containing a carbamoyl group at this position (C-13). The gene responsible for the formation of the carbamoyl group originally identified in *C. raciborskii* T3 [21], is the 1839 bp long O-carbamoyltransferase denoted *sxtI*. The *sxtI* gene homolog from *L. wollei* was only 1071 bp in length. Recently, Kellmann et al. [27] have shown that the *L. wollei sxtI* first 912 base-pairs had 92.9% identity to *sxtI* from *C. raciborskii* T3, base-pairs 913 to 1059 and 1177 to 1189 (*C. raciborksii* T3 numbering) were deleted, and the 3′-end was truncated after base-pair 1227. As a result of these deletions the partial *L. wollei sxtI* translated enzyme does not contain the putative catalytic site GPRALGGRS [27]. It is therefore, most probably inactive. The apparent truncation of *sxtI* might be the result of a partial gene deletion event or alternatively by the insertion of a gene, denoted *sxtACT* (Figure 2). *sxtACT* codes for an enzyme similar to an O-acyltrasferase belonging to the CoA-dependent acyltransferase superfamily. A member of this family, Deacetylvindoline 4-O-acetyltransferase (EC:2.3.1.107) catalyzes the last step in vindoline biosynthesis [28]. It is also possible that *sxtACT* could be an ancient sxt gene that has been lost in the other cyanobacterial lineages. Irrespective of whether *sxtI* and *sxtACT* had been recently acquired or lost from the PST biosynthesis clusters, genetic rearrangement has resulted in the absence of carbamoylated saxitoxins and the presence of the novel C-13 acetylated saxitoxin analogs in *L. wollei*. Furthermore, the three novel genes identified in the *L. wollei* saxitoxin gene cluster, namely *sxtACT*, *sxtSUL* and *sxtdiox*, appear to have been inserted as one locus, thereby conferring the ability to produce analogs **1-6** in *L. wollei*.

*sxtL*, a gene which is not present in the *L. wollei* PST gene cluster codes for an enzyme similar to a GDSL lipase, which has been proposed to catalyze the hydrolytic cleavage of the carbamoyl group from STX analogs in *C. raciborskii* T3. The absence of an active carbamoyltransferase in *L. wollei* would explain the lack of carbamoylated saxitoxin derivatives produced by *L. wollei.* In this modified genetic background, that is, without an active *sxtI* homolog, and therefore no carbamoylated saxitoxins, the lack of a decarbamoylating enzyme, SxtL, seems evolutionally logical.

*L. wollei* synthesizes a new group of derivatives that contain only one carbinol residue at C-12 (denoted **1**,**4** and **6** Figure 1), whereas all other previously identified saxitoxins have a hydrated ketone function at that position, a so called geminal hydroxyl [7]. Only one epimer, the b-H form, of these 12-dihydro derivatives has been identified in *L. wollei*, and this is therefore a specific enzymatic step in the biosynthesis of these compounds [7]. The *L. wollei* gene cluster encodes three distinct dioxygenases, denoted *sxtH*, *sxtT* and *sxtdiox*, which are most similar to phenylpropionate dioxygenases. These enzymes contain two domains; an N-terminal Rieske domain with an [2Fe-2S] cluster and a C-terminal catalytic domain with a mononuclear Fe(II) binding site [29]. *sxtT* and *sxtH,* originally identified in *C. raciborskii* T3, are putatively involved in the hydroxylation of the terminal diol in saxitoxin biosynthesis [21]. However, *sxtdiox* is unique to the *L. wollei* and *R. brookii* D9 PST gene clusters. In a recent detailed evolutionary analysis of the *sxt* genes, Murray et al. [30] found that the *sxtH/T* genes form a monophyletic *sxt* clade, which is under positive selection and has undergone significant intragenomic or intraspecific recombination. *sxtH* has likely duplicated in *L. wollei*, or the ancestral strain, giving rise to *sxtT*, and duplicated again to give rise to sxtdiox in *R. brookii* D9 and *L. wollei* [30].

**Figure 3:**
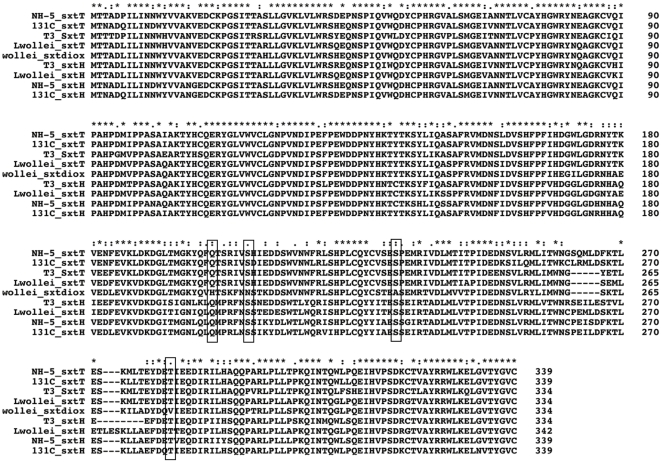
Amino acid alignment of the dioxygenases encoded by the various saxitoxin clusters. The conserved regions that are modified in *sxtdiox* are boxed.

Analysis of the dioxygenase protein alignment revealed that three positions at the C-terminus of the enzyme, conserved in the saxitoxin dioxygenases, are modified in *sxtdiox* (Figure 3). As the mechanism of catalysis is unknown in this enzyme family it is not possible to determine the effect of these alterations [31]. However, we propose that that *sxtdiox* carries out the hydroxylation of C-12 in the saxitoxin derivatives, **1**, **4**, **6**, thereby forming the novel 12-dihydro-derivatives. It will be of biochemical significance to confirm this novel activity by heterologous expression and characterization of this enzyme.

*L. wollei* also produces O-sulfated saxitoxin analogs (dcGTX2/3, **1**,**2**,**3**) and the *L. wollei* gene cluster encodes two sulfotransferases (*sxtN1* and *sxtN2*) homologous to *sxtN* identified in *C. raciborskii* T3 [21], which presumably transfers a sulfate group to O-22 forming dcGTX2/3. *sxtN1* and *sxtN2* are highly similar, with a nucleotide sequence identity of 97%. However, *sxtN2* contains a stop codon in its ORF, and therefore is most probably inactive and possibly represents a gene duplication event. The *L. wollei* saxitoxin gene cluster also contains another gene bioinformatically identified as a PAPS dependent sulfotransferase, namely *sxtSUL*, which is unique to the *L. wollei* and *R. brookii* D9 PST gene clusters. We postulate that due to its uniqueness in this gene cluster and its homology to other characterized sulfotransferases, that sxtSUL might sulfate the novel saxitoxin analogs produced by *L. wollei* (**5**,**6**) forming the saxitoxin analogs **1**,**2**,**3**, though its involvement in the formation of the GTX toxins cannot be excluded.

Surprisingly, the *L. wollei* PST gene cluster contains the PST tailoring enzyme *sxtX*, a gene similar to cephalosporin hydroxylase, and presumably responsible for N-1 hydroxylation of STX, thereby converting STX to neoSTX [21], [22]. This finding is not in agreement with the analysis of Onodera et al. [7], which did not reveal any N-hydroxylated PST derivatives in the *L. wollei* isolate analyzed. This discrepancy might be attributed to low levels of N-hydroxylated derivatives in the analyzed sample, bellow the limit of detection of methods used, or alternatively might be the result of novel STX derivatives which were not elucidated with the methods used. On the other hand, the *L. wollei* biomass used in our study, although from the same source (Guntersville reservoir), might be of a dissimilar strain with a different genetic background to the sample analyzed by Onodera et al. [7], and therefore might have a distinct toxin profile. A further explanation would include a different catalytic activity for *sxtX* than was previously proposed, clarifying its actual activity will require the heterologous expression of *sxtX* to determine its catalytic activity and natural substrates. In order to reconcile these apparent discrepancies a culturable strain of *L. wollei* needs to be analyzed for the presence of PST biosynthesis genes as well as PST derivatives produced, though this was not possible at the time of this study as the available freeze-dried *L. wollei* biomass was not suitable for reliable PST composition analysis.

Recent studies into the kinetics of PST accumulation in cyanobacterial cells and growth media, suggest an active transport mechanism [32], [33]. The recently identified saxitoxin gene clusters [21], [22] also contain genes coding for Multidrug And Toxic compound Extrusion (MATE) proteins of the NorM family. The *L. wollei* saxitoxin gene cluster contains three genes belonging to this family, *sxtM1*, *sxtM2* and *sxtM3*. Furthermore, the *L. wollei* PST cluster also contains a gene, denoted *sxtPER*, which is most similar to permeases of the drug and metabolite transporter (DMT) family. This gene is also present in *A. circinalis* 131C and *Aph. sp.* NH-5 [22] but absent in the gene cluster of *C. raciborskii* T3 [21]. The observed multitude of transporters present in the *L. wollei* PST gene cluster might be attributed to the variety and uniqueness of PST analogs produced by this species, or conversely to an adaptation of its transport mechanism to pressures in its environmental niche, requiring increased extracellular transport of PSTs.

Curiously, *orf3*, which is located between *sxtV* and *sxtU* also shows high similarity to *sxtPER*, though it is truncated and presumed inactive, and might be a result of a further gene duplication/recombination event. In addition, there are further indications of previous gene duplication and deletion events in the *L. wollei* PST biosynthesis gene cluster. *Orf 5*, which is located 5 prime to *sxtM1*, and contains a short truncated sequence which is highly similar to *sxtH*, a hydroxylase putatively involved in the hydroxylation of C-12 of saxitoxin. This fragment might be the result of the gene duplication event that formed the *L.wollei* unique transporters *SxtM1* and *sxtM2*, as the intact copy of *sxtH* is also 5 prime and adjacent to *sxtM2*.

### Evolution and distribution of PST biosynthesis in Lyngbya

Lateral gene transfer (LGT) and gene deletions are probable explanations for the sporadic distribution of saxitoxin producers among the different cyanobacterial species and across kingdoms [22], [27], [30]. The variations observed in the structure and gene content of the saxitoxin gene cluster in *L. wollei* may be a direct result of its mobility via the recombination events that are inherent to transposition. This is further supported by the presence of multiple copies of transposases identified in many secondary metabolite gene clusters, including those of other cyanobacterial toxins such as cylindrospermopsin, microcystin, nodularin and saxitoxin [34], [35], [36], [37,]. Interestingly, a small gene fragment denoted *orf 2*, which is located between *sxtA* and *sxtB* shows high similarity to the gene *cyrB* of *C. raciborskii* AWT205, which is involved in the biosynthesis of the cyanotoxin cylindrospermopsin [35]. This finding could indicate that part of the cylindrospermopsin biosynthesis gene cluster was present in this cyanobacterium and was lost through gene deletion and/or recombination event, while also possibly acquiring the PST biosynthesis genes. Curiously, *L. wollei* seems to present a geographical segregation in toxin production, as isolates from North America (such as the sample analyzed in this study) have so far only been shown to produce PSTs, while Australian isolates have only been shown to produce cylindrospermopsin [7], [38], [39]. A similar geographical distribution of toxicity is seen in the genera *Cylindrospermopsis*, that produce PSTs in South America and cylindrospermopsin in Europe and Australia [17], [40], [41], [42], and *Anabaena*, which only produce PSTs in Australia [15], [23], [24]. These results further support the view that LGT and gene deletions contribute to the evolution and distribution of cyanobacterial toxin biosynthesis genes. In agreement with these findings, recent phylogenetic analysis of cyanobacterial PST genes [30], [37] has indicated that the PST genes are of ancient origin, with a complex history involving horizontal gene transfer from different sources, whereby the ability to produce PST was subsequently lost multiple times in non-PST producing cyanobacteria.

### Biosynthesis of PST congeners

**Figure 4:**
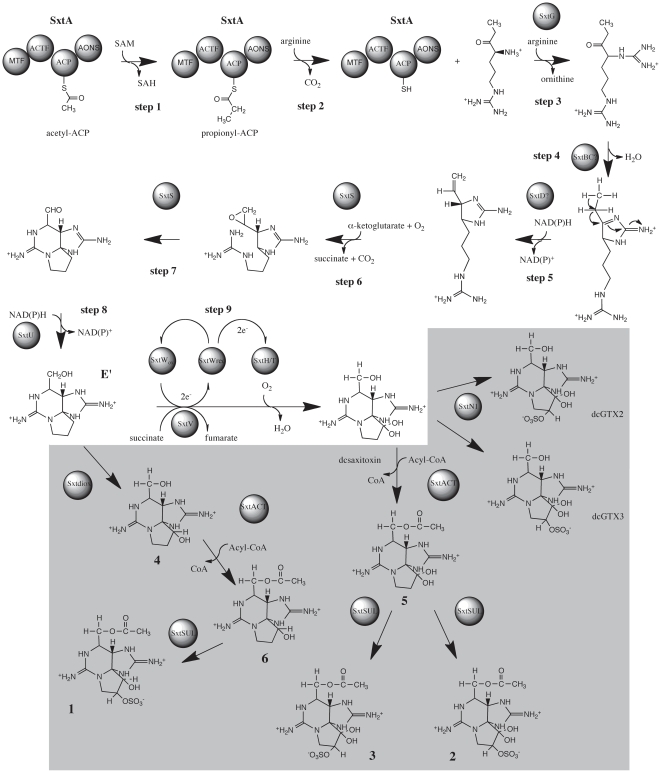
Proposed biosynthetic pathway for the PSTs produced by *Lyngbya wollei*. The gray shaded area highlights the steps that are unique to *L. wollei*.

Based on the *in silico* analysis of the *L. wollei* PST gene cluster, the recently elucidated putative saxitoxin biosynthetic pathway [21], [22] has been correspondingly modified (Figure 4). In *L. wollei*, we propose that the biosynthetic steps leading up to the formation of intermediate E' are identical to the pathway described for *C.* raciborskii, *A. circinalis* and *Aph.* sp. [21], [22] (steps 1- 8 Figure 4). Thereafter, sxtH and sxtT, each coding a terminal oxygenase subunit of bacterial phenyl-propionate and related ring-hydroxylating dioxygenases, catalyze the consecutive hydroxylation of C-12, forming dcSTX (Figure 4 step 9). Unique to *L.wollei* are the proposed novel tailoring reactions leading to the formation of *Lyngbya*-specific saxitoxin isoforms (Figure 1,4). dcGTX2 and dcGTX3 are putatively formed by a sulfur transfer onto O-11 of dcSTX catalyzed by sxtN1 (sulfotransferase). sxtACT, an acyl-CoA dependant acyltransferase moves an acyl group onto the hydroxyl at C-13 of dcSTX resulting in analog **5**. The sulfotransfer onto analog **5**, catalyzed by SxtSUL, synthesizes analogs **2** and **3**. The sxtdiox catalyzed, single hydroxylation of the biosynthetic intermediate produced by step 8 (Figure 4) forms analog **4**. An acyltransfer onto the analog **4** C-13 hydroxyl, via the action of sxtACT results in the formation of analog **6**, which can then be sulfated by SxtSUL to form analog **1**.

As noted by Onodera et al. [7], the lack of an 11-a-O-sulfate epimer of saxitoxin analog **1** (Figure 1) in *L. wollei* indicated that the reduction of C-12 occurs prior to the introduction of 11-O-sulfate in a stereo specific manner from the b-side of the molecule [7]. This predicted biosynthesis pathway should be confirmed by future heterologous expression of the enzymes and molecular intermediate analysis by mass-spectrometry.

Analysis of saxitoxin gene clusters in the numerous and divergent producing organisms provides insights into the biosynthetic machinery and the variations that give rise to different toxin profiles. This in turn enables the construction of hypotheses regarding the role of the genes present, which will direct validation by recombinant expression in heterologous hosts. A case in point are the genetic rearrangements identified in the *L. wollei* PST biosynthesis gene cluster, which include a putative truncation and inactivation of the carbamoyltransferase gene (*sxtI*), coupled with the presence of an acyltransferase gene (*sxtACT*), a novel sulfotransferase (*sxtSUL*) and a dioxygenase (*sxtdiox*) adjacent to each other. This observed genetic variance presumably resulted in a gene cluster that does not confer the ability to produce carbamoylated saxitoxin derivatives. The new family of saxitoxin analogs made possible by this genetic rearrangement, contain an acetate group at the same position (C-13), as well as a carbinol at C-12, and have shown reduced neurotoxicity [7]. The deletion identified in the *L. wollei sxtI* gene further confirms the putative role assigned to sxtI as a carbamoyltransferase, based on bioinformatic analysis of the gene [27] and *in vitro* biosynthesis studies [43] that show carbamoylphosphate, the natural substrate for carbamoyltransferases, to be a precursor for saxitoxin biosynthesis. This example of natural combinatorial biosynthesis indicates the ability to produce unnatural compounds from alkaloid biosynthetic pathways, enabling the production of novel biologically active compounds. The *in silico* functional assignment of *sxt* genes in *L. wollei*, has enabled the construction of a putative PST biosynthetic pathway, thereby elucidating the production of the novel saxitoxin analogs by this organism (Figure 1). The novel PST tailoring genes identified in *L. wollei*, add to the catalytic collective available for the growing field of combinatorial biosynthesis.

The gene sequence data presented in this study will enable future investigations into the regulation of PST biosynthesis gene expression in this organism using techniques such as promoter analysis and real-time PCR whereby providing insights into the physiological roles of the PSTs and furthering our ability to predict and prevent the formation of harmful algal blooms. The availability of more saxitoxin biosynthesis gene sequences will enable better monitoring of algal blooms for water authorities, including PCR-based early warning systems such as qPCR and toxin gene specific PCRs. This work may also facilitate the identification of the genes involved in saxitoxin biosynthesis in dinoflagellates, which are the cause of human mortality and great economic damage to the shellfish and tourism industries.

## Materials and Methods

### Cyanobacterial sampling

*Lyngbya wollei* (Farlow ex Gomont) was originally isolated from Guntersville Reservoir (Guntersville, AL) and its taxonomy and toxicity subsequently determined [16]. A field sample of a *Lyngbya wollei* unialgal bloom from Guntersville Reservoir was freeze-dried, stored at −20°C and used for the subsequent DNA isolation.

### DNA extraction

Total genomic DNA was extracted from freeze-dried cyanobacterial cells using the Mo Bio PowerPlant DNA isolation kit (Carlsbad) in accordance with the manufacturers instructions. Genomic DNA was stored at −20°C.

### 16S rRNA gene analysis

Genomic DNA isolated from *L. wollei* was amplified using the 16S rRNA gene primers 27F and 809R as previously described [44], and deposited in GenBank under accession number EU603708.

### Saxitoxin gene amplification

We have recently identified the gene clusters responsible for the biosynthesis of saxitoxin in the cyanobacteria *C. raciborskii* T3, *A. circinalis* AWQC131C, and *Aph. sp.* NH-5 [21], [22]. Based on this sequence information, amino acid alignments of two highly similar enzymes in the saxitoxin gene clusters, namely *sxtT* and *sxtH*, which are putatively involved in the formation of the terminal diol at C-12 in saxitoxin [21], were created. Degenerate PCR primers targeting *sxtT* and *sxtH* used in this study (DioxF 5′ CCNGARTGGGAYGAYCCNAAYTA 3′ DioxR 5′ ATRTCYTGYTCDATNGTYTCRTC 3′) were designed *in silico* from sequence alignments produced using ClustalX [45]. Degenerate PCR was performed in 20 µL reaction volumes containing 1× *Taq* polymerase buffer, 2.5 mM MgCl_2_, 0.2 mM deoxynucleotide triphosphates, 25 pmol each of the forward and reverse primers, 50 ng of genomic DNA and 0.2 U of *Taq* polymerase (Fischer Biotech). Thermal cycling was performed in a GeneAmp PCR System 2400 Thermal cycler (Perkin Elmer Corporation). Cycling began with a denaturing step at 94°C for 4 min followed by 35 cycles of denaturation at 94°C for 10 s, primer annealing at 50°C for 30 s and a DNA strand extension at 72°C for 45 s. Amplification was completed by a final extension step at 72°C for 5 min.

Amplified DNA was analyzed by agarose gel electrophoresis in TAE buffer (40 mM Tris-acetate, 1 mM EDTA, pH 7.8), and visualized by UV transillumination after staining with ethidium bromide (0.5 µg · mL). Where multiple amplicons were detected during the gel electrophoresis, single amplicons were excised from the gels and purified using the Promega Wizard® SV Gel and PCR Clean-Up, prior to sequencing.

### Gene cloning

Clone libraries were created using the pGemT-easy cloning kit (Promega) in accordance with the manufacturers instructions.

*DNA sequencing.* Automated DNA sequencing was performed using the PRISM Big Dye cycle sequencing system and a model 373 sequencer (Applied Biosystems).

### Gene walking

Characterization of unknown regions of DNA flanking the putative saxitoxin biosynthesis genes, *sxtT* and *sxtH,* in *L. wollei* was performed using an adaptor-mediated PCR as previously described [21], [22], [34]. PCRs were performed in 20 µL reaction volumes containing 1× *Taq* polymerase buffer, 2.5 mM MgCl_2_, 0.2 mM deoxynucleotide triphosphates, 10 pmol each of the forward and reverse primers, between 10 and 100 ng of genomic DNA and 0.5 U of a mixture of 10:1 *Taq* polymerase/PFU (Fischer Biotech). Thermal cycling was performed in a GeneAmp PCR System 2400 Thermal cycler (Perkin Elmer Corporation). Cycling began with a denaturing step at 94°C for 2 min followed by 30 cycles of denaturation at 94°C for 10 sec, primer annealing between 55°C and 65°C for 20 sec and a DNA strand extension at 72°C for 3 min. Amplification was completed by a final extension step at 72°C for 7 min.

### Bioinformatic analysis

Sequence data were analyzed using ABI Prism-Autoassembler software, while identity/similarity values to other translated sequences were determined using BLAST in conjunction with the National Center for Biotechnology Information (NIH, Bethesda, MD). Fugue blast (http://www-cryst.bioc.cam.ac.uk/fugue/) was used to identify distant homologs via sequence-structure comparisons. The gene clusters were assembled using the software package Phred, Phrap, and Consed (http://www.phrap.org/phredphrapconsed.html), open reading frames were manually identified. Alignments were performed using ClustalX [45].

### Nucleotide sequence accession number

The *L. wollei* PST gene cluster sequences were submitted to GenBank and are available under the accession number EU603711.
